# Protein Secretion Is Required for Pregnancy-Associated Plasma Protein-A to Promote Lung Cancer Growth *In Vivo*

**DOI:** 10.1371/journal.pone.0048799

**Published:** 2012-11-09

**Authors:** Hong Pan, Sayaka Hanada, Jun Zhao, Li Mao, Mark Zhi-Qing Ma

**Affiliations:** 1 Department of Oncology and Diagnostic Sciences, University of Maryland School of Dentistry, Baltimore, Maryland, United States of America; 2 Experimental Therapeutic Program, Greenbaum Cancer Center, University of Maryland, Baltimore, Maryland, United States of America; 3 Thoracic Surgery Department, Affiliated Tumor Hospital of Guangxi University, Nanning, Guangxi Province, People's Republic of China; 4 Department of Thoracic Medical Oncology, Peking University School of Oncology, Beijing Cancer Hospital, Beijing Cancer Institute for Cancer Research, Haidian District, Beijing, People's Republic of China; University Magna Graecia, Italy

## Abstract

Pregnancy-associated plasma protein-A (PAPPA) has been reported to regulate the activity of insulin-like growth factor (IGF) signal pathway through proteolytic degradation of IGF binding proteins (IGFBPs) thereby increasing the local concentration of free IGFs available to receptors. In this study we found that PAPPA is secreted from two out of seven lung cancer cell lines examined. None of immortalized normal bronchial epithelial cells (HBE) tested secrets PAPPA. There is no correlation between expression level and secretion of PAPPA in these cells. A cell line over-expressing PAPPA accompanied with secretion shows no notable changes in proliferation under cell culture conditions *in vitro*, but displays significantly augmentation of tumor growth *in vivo* in a xenograft model. In contrast, a cell line over-expressing PAPPA without secretion exhibits reduction of tumor growth both *in vitro* and *in vivo*. Down-regulation of PAPPA expression and secretion by RNAi knockdown decreases tumor growth after implanted *in vivo*. The tumor promoting activity of PAPPA appears to be mediated mainly through augmentation of the IGF signaling pathway as indicated by notable increases in downstream Akt kinase phosphorylation in tumor samples. Our results indicate that PAPPA secretion may play an important role in lung cancer growth and progression.

## Introduction

Insulin-like growth factors (IGFs) play important roles in many biological processes such as cell growth, transformation, differentiation, survival and migration [1], [2]. In addition to their critical roles in developmental biology as evident in genetic null mutations [3], [4], [5], IGFs are clearly involved in the regulation of tumor formation and progression [6], [7]. It is well known that serum levels of IGF-1 correlate with cancer incidence in humans [8], [9]. Mouse embryonic fibroblasts with a null mutation of *Ifgf1r* are resistant to induction of cell transformation [10]. At the molecular level, IGFs bind to the receptor tyrosine kinases (IGF1R, IR-A) to activate multiple intracellular signaling pathways, including phosphatidylinositide-3′-kinase (PI3K)/Akt and mitogen-activated protein kinase (MAPK) signaling cascades [11], [12]. However, the access of IGFs to their receptors is tightly controlled by IGF-binding proteins (IGFBPs) which bind to IGFs with a higher affinity than IGF1R [13]. Degradation of IGFBPs increases free active IGFs available to the receptors in the extracellular microenvironment and thus enhances IGFs activity [14]. A diverse group of proteases including plasmin, matrix metalloproteases (MMP-1, -2 and -3), cathepsin D and prostate-specific antigen (PSA) have been reported to participate in proteolysis of IGFBPs with different potency and specificity [15].

Pregnancy-associated plasma protein-A (PAPPA) is another protease that has been reported to cleave IGFBP4 [16]. PAPPA was initially found in the placenta and reproductive tissues, and has been suggested as a biomarker for pregnancy with genetic abnormality [17]. Recently the proteolytic activity of PAPPA has been identified in normal human fibroblasts, cultured osteoblasts [18], [19], vascular smooth muscle cells [20] and ovarian granulosa cells [21]. In line with these findings, increased expression of PAPPA has been found *in vivo* in active atherosclerotic plaques in human coronary arteries [22] and wound healing of human skin [23]. Thus, PAPPA participates in regulation of IGFs mediated pathophysiological processes of these diseases. PAPPA is also found to cleave and inactivate IGFBP2, IGFBP3 and IGFBP5 [24].

Several lines of evidences indicate that PAPPA is implicated in tumor formation. PAPPA gene is localized in a chromosomal region associated with high frequency of loss of heterozygosity in ovarian tumors. Most ovarian cancer cell lines and primary tumors show partial or complete loss of expression of PAPPA [25]. PAPPA expression was shown to be consistently high in normal ovarian samples and was suppressed by SV40 large T antigen [25], [26]. On the contrary, mice bearing a null mutation of the PAPPA gene live longer and show less incidence of tumor formation during their lifetime [27]. In addition, serum PAPPA level has been reported to be increased in patients with lung cancer in comparison with healthy subjects [28]. In the view of the controversial roles of PAPPA reported in cancer development, we decided to over-express PAPPA in lung cancer cell lines to evaluate its role on tumor growth and progression. Here we report that ectopic over-expression of PAPPA in H1299 lung cancer cells promotes tumor growth *in vivo* in a xenograft model while down regulation of endogenous PAPPA in A549 lung cancer cells decreases tumor growth. Tumor growth rate is associated with PAPPA secretion. Tumors from PAPPA over-expressing H1299 cells exhibited increased numbers of mitotic cells and a reduction in apoptosis. Signaling pathway analysis showed elevated Akt signaling. Our results suggest that secreted PAPPA in cancer cells could promote tumor development through potentiating the IGF signaling pathway.

## Materials and Methods

Reagents were obtained from the following suppliers: Cell lines H1299, A549, H460, H596, H1792, H1944 and H522 were originally purchased from ATCC. HBE1, HBE2 and HBE4 were obtained from John Minna (University of Texas Southwestern Medical Center, Dallas), each representing HBEC1, HBEC2 and HBEC4 immortalized with Cdk4 and hTERT, respectively [29]. Origin of reagents used in experiments is listed as follows: Human PAPPA full length cDNA clone (ATCC, Item No: 10625309); Mouse monoclonal anti-PAPPA antibody (Novus, Littleton CO); IGF2 protein, IGFBP4 protein and Rabbit polyclonal anti-IGFBP4 antibody (Abcam, Cambridge, MA); Phospho-p44/42 MAPK rabbit mAb, p44/42 MAPK rabbit mAb, Phospho-Akt rabbit mAb, Akt (pan) rabbit mAb, β-tubulin antibody (Cell Signaling, Danvers, MA); Anti-mouse IgG, Anti-rabbit IgG (Thermo Scientific, Rockford, IL); Biotinylated anti-rabbit IgG, Biotinylated anti-mouse IgG, (Vector Lab, Burlingame, CA); Recombinant human epidermal growth factor (EGF), Recombinant human transforming growth factor-β1 (TGF- β1) (Invitrogen, Camarillo, CA); Recombinant human insulin-like growth factor I (IGF1) (R&D System, Minneapolis, MN); CellTiter-Blue (Promega, Madison, WI); Anti-phospho histone H3 (Millipore, Temecula, CA); RIPA Buffer (Sigma, Saint Louis, MI); PAPPA ELISA kit (Diagnostic Systems Lab, Webster, TX); Protease inhibitor cocktail tablets (Roche Applied Science, Mannheim, Germany)

### Cell culture

H1299, H460, H596, H1792, H1944 and H522 cell lines were maintained in RPMI1640 and A549 cell cultures were maintained in DMEM. Both PRMI1640 and DMEM were supplemented with 10% fetal bovine serum (FBS) (Atlanta, Lawrenceville, GA), L-glutamine (4 mM), penicillin (100 U/ml), and streptomycin (100 µg/ml), all from Invitrogen. HBE1, HBE2 and HBE4 were maintained in complete keratinocyte serum free medium (SFM) (Invitrogen, Carlsbad, CA). Cells were cultured in a humidified tissue culture incubator at 37 C, 5% CO_2_. Serum-free cell conditioned media (CM) was prepared from monolayer cultures at 80–100% confluence. Serum-containing media was aspirated and the monolayers were washed three times with phosphate-buffered saline. Complete keratinocyte-SFM was added and incubated for 24 hours. At harvest, CM was transferred to a centrifuge tube and centrifuged at 2,500 rpm for 10 min at 4°C before freezing in small aliquots at −20°C until used in assays. For whole cell extract (CE), medium was removed and washed three times in PBS and lysed in RIPA buffer (Sigma-Aldrich, St. Louis, MO) containing protease and phosphatase inhibitor cocktail tablets (Roche Applied Science, Indianapolis, IN) and centrifuged at 12,500 rpm for 5 min. This supernatant was used for assays.

### Expression analysis of cancer cell lines by real-time PCR

The RNeasy Mini kit (Qiagen, Valencia, CA) was used to prepare total RNA from cell culture. Total RNA was treated with DNase according to the manufacturer's instructions (Qiagen). Synthesis of cDNA was set up using TaqMan RT-PCR reagents from Applied Biosystems (Life Technologies, Grand Island, NY). PAPPA gene expression was determined by TaqMan gene expression assay with a 7900HT Real-Time PCR System detector (Applied Biosystems). The specific primers for amplification of target genes and specific probe for detect amplification were designed by Applied Biosystems (Cat#: Hs00361692_m1). Human PPIA (Cyclophilin A) was used as an internal control for normalization of gene expression (Cat#: 4333763F, Applied Biosystems).

### Generation of lung cancer cell lines stably over-expressing PAPPA or down regulation of endogenous PAPPA expression

Human PAPPA full length cDNA (purchased from ATCC, Item No: 10625309) was released from pBluescript vector by SfiI (filled)-XbaI digestion and subcloned into EcoRV and XbaI sites of pCR3.1 expression vector (Invitrogen) to construct PAPPA expression vector. The expression vector was verified by sequencing. H1299 cells transfected with either PAPPA expression vector or empty vector were selected under selection medium contain 0.5 mg/ml G418 (Sigma). G418 resistant clones were tested for the expression of PAPPA and then expanded to generate H1299 PAPPA over-expression line (H1299/PAPPAov) and empty vector control cell line (H1299/pCR3.1). For PAPPA knockdown experiments, lentiviral particles #81 (Cat#: VGH5523-101126912, Clone ID: V3LHS_355181), #82 (Cat#: VGH5523-101130068, Clone ID: V3LHS_355182), #83 (Cat#: VGH5523-101132535, Clone ID: V3LHS_355183) that contain shRNAmir to PAPPA and non-silencing shRNAmir (Cat#: RHS4348) were purchased from Thermo Scientific (Lafayette, CO) and utilized to infect A549 cancer cells. The mature sense sequences of these clones are listed as follows: V3LHS_355181: CGACAAGAAGTCTCCTTCA; V3LHS_355182: AGAGCCTACTTGGATGTTA; V3LHS_355183: GGGCAGTTCATGAAGCTCT; Non-silencing: TCTCGCTTGGGCGAGAGTA. Forty eight hours after infection, cells were selected in selection medium containing 2 µg/ml puromycin (Invitrogen). Clones were examined by real-time PCR for potency of PAPPA mRNA knockdown and expanded to generate PAPPA knockdown cell line (A549/PAPPAkd) and non-silencing control cell line (A549/sc).

### PAPPA ELISA

Conditioned medium (CM) and whole cell extract (CE) were prepared as described above. PAPPA levels were measured using an Ultrasensitive PAPPA ELISA kit obtained from Diagnostic Systems Laboratories according to the manufacturer's instructions. Minimum sensitivity is 0.24 mIU/L, with intra- and inter-assay coefficients of variation of 4.7 and 4.2%, respectively.

### Measurement of IGFBP proteolytic activity

The proteolytic activity of PAPPA was analyzed by incubating serum-free conditioned medium (CM) with substrate protein IGFBP4. Briefly, replicate reactions were set up by mixing 25 µl CM with 40 ng IGFBP4 substrate in the presence or absence of IGF2 (75 nM final concentration) followed by incubation at 37°C for 4 hours. Intact and cleaved protein of IGFBP4 in reaction mixtures were then separated by non-reducing 10% SDS-PAGE and visualized by Western Blot analysis using polyclonal anti-IGFBP4 antibody (Abcam).

### Tumor xenograft experiments

Four-week old female severe combined immune deficiency (SCID/NOD) mice or nu/nu athymic mice (Charles River) were used for this study, and all mice were kept under specific pathogen-free conditions. 5×10^6^ of H1299/PAPPAov, H1792/PAPPAov or its empty vector control cells (H1299/pCR3.1 or H1792/pCR3.1) were suspended in 0.1 ml of 1× PBS containing 50% of matrigel and injected subcutaneously into five mice per group using 26 gauge needles and allowed to propagate to form palpable tumors. Tumors were monitored and then measured twice a week in three dimensions using a caliper. Similarly, PAPPA knockdown A549 cells line A549/PAPPAkd and non-silencing control A549sc were expanded in culture. 1×10^6^ cells suspended in 0.1 ml 1× PBS were injected subcutaneously into four week old nu/nu athymic mice. The growing tumors were monitored and measured twice a week as described above. Tumor volumes were calculated using the formula (π/6)×length (*L*)×width (*W*)×height (H) of tumors where *L* and *W* represent the longest and shortest tumor dimensions, respectively. The mice were euthanized in the same manner when the largest tumor reached the study endpoint defined by a tumor burden of 10% of mouse body weight.

### Ethics Statement

All mice were handled according to the Guide for the Care and Use of Laboratory Animals. All animal experiments were carried out following procedures approved by the Institutional Animal Care and Use Committee at the University of Maryland School of Dentistry. The named institutional review board or ethics committee specifically approved this study.

### Western Blot Analysis

Protein from whole cell extractions was quantified using the BCA assay (Pierce). A total of 25 µg of protein was fractionated by NuPAGE 10% Bis-Tris gel (Invitrogen) and transferred onto a polyvinylidene difluoride (PVD) membrane by iBlot gel transfer system (Invitrogen). The membrane was blocked in TBST (10 mMTris-HCl, pH 7.4, 100 mM NaCl, 0.1% (v/v) Tween-20) containing 5% (w/v) non-fat powdered milk for one hour and incubated overnight at 4°C with primary antibodies (1∶1000) either in TBST/1% (w/v) non-fat powdered milk or in TBST/1% (w/v) BSA (bovine serum albumin). Membranes were washed six times with TBST, incubated with 1∶5000 horseradish peroxidase conjugated anti-mouse or anti-rabbit IgG antibody in TBST/1% (w/v) non-fat powdered milk for one hour at room temperature. After another six washes in TBST, antibody complexes were detected using enhanced chemiluminescence (Amersham Biosciences). Molecular weights were estimated using a prestained full-rage rainbow molecular weight markers (GE Healthcare).

### Immunohistochemical Staining

Paraffin-embedded, 5 µm thick tissue sections from tumor tissues were used for immunohistochemical staining. Briefly, sections were deparaffinized in a series of xylene baths and then rehydrated using a graded alcohol series. The sections were then incubated in citrate buffer (pH 9) for 15 min under microwave radiation in order for antigen retrieval. After blocking with normal blocking buffer (Vectastain kit) for 30 min, sections were incubated overnight at 4°C with primary antibodies (Phospho-histone H3 mAb: 1∶000; Phospho-Akt mAb: 1∶100; Phospho-p44/42 MAPK mAb: 1∶100). The sections were then processed using standard avidin-biotin immunochemical techniques (ABC) according to the manufacturer's recommendations (Vector Laboratories). Diamnobenzidine was used as a chromogen and hematoxylin was used for counterstaining.

## Results

### Secretion of functional active PAPPA from lung cancer cells

**Figure 1:**
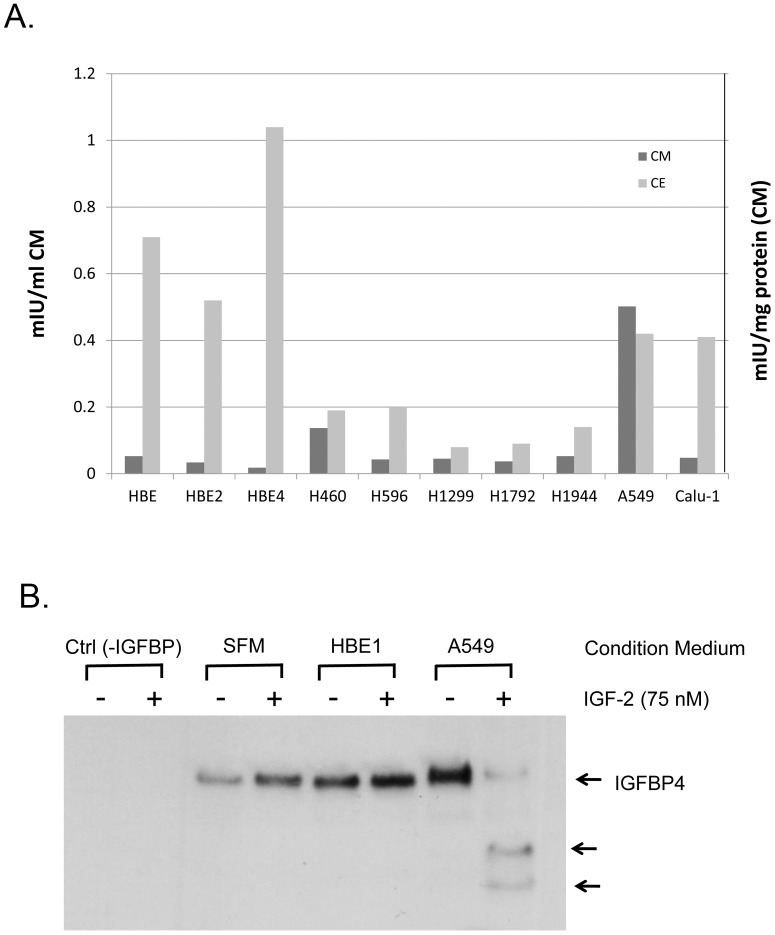
PAPPA expression and secretion in lung cancer cell lines. (A)PAPPA protein concentration in different lung cancer cell lines (CE) and conditioned medium (CM). Data shown are the average of two independent assays in duplicate. (B) IGF dependent protease activity in conditioned medium of A549 lung cancer cells. Measurement of IGFBP proteolytic activity is carried out as described in Materials and Methods. Arrows indicate intact IGFBP4 and 18 kD and 14 kD proteolytic fragments.

We first tested the hypothesis whether the increased plasma concentration of PAPPA observed in lung cancer patients [28] is due to secretion of PAPPA directly from lung cancer cells. We examined protein content and secretion of PAPPA in several commonly used non-small cell lung cancer (NSCLC) cell lines in comparison with immortalized normal human bronchial epithelial (HBE) cell lines. PAPPA content in cancer cell lines is comparable (in cases of A549 and Calu-1) or less (in case of H460, H596, H1299, H1792 and H19444) than HBE cell lines in general. However, PAPPA secretion from A549 and H460 is significantly more than HBE cells as evidenced by significant high levels of PAPPA in conditioned medium from these two cell lines. The results suggest PAPPA protein secretion may be different between HBE cells and A549, although total protein levels of PAPPA are similar (Fig. 1A). Thus the secreted form of PAPPA from cancer cells may contribute to the growth and progression of some types of lung cancer.

To further address if secreted PAPPA is functionally active, the protease activity of PAPPA was examined using IGFBP4 as substrate in conditioned medium from HBE1 and A549. As shown in Figure 1B, conditioned medium from A549 could effectively degrade IGFBP4 in the presence of IGF2, indicating that PAPPA secreted from A549 is functionally active. PAPPA in HBE1 is neither secretive nor functional because no IGFBP4 degradation was observed in the conditioned medium from HBE1 (Fig. 1B). Taken together, these results suggested that the ability of cancer cells to secret PAPPA rather than cellular PAPPA content is associated with tumor growth and progression. Functional assay of PAPPA proteolytic activity on IGF2 dependent IGFBP4 degradation is specific, sensitive and reliable. We used this assay thereafter as means to detect the presence of PAPPA serum free medium as reported by others [26], [27], [30].

### Reduction of PAPPA secretion decreases tumor growth in vivo in a xenograft model, but not in vitro in cell culture

**Figure 2:**
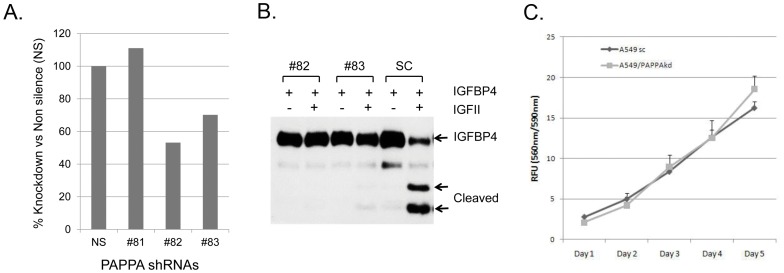
PAPPA knockdown in A549 cells lines. A549 cells were transduced with lentiviral particles containing shRNAmir (#81 #82 and #83) to PAPPA and non-silencing shRNAmir (SC) and selected with puromycin. (A) PAPPA mRNA levels from these cell lines determined by real-time PCR quantification and depicted as relative level to non-silencing control. (B) IGF dependent protease activity in conditioned medium from PAPPA knockdown A549 cells lines. (C) Growth curve of PAPPA knockdown A549 cells line #82 and non-silencing control line.

**Figure 3:**
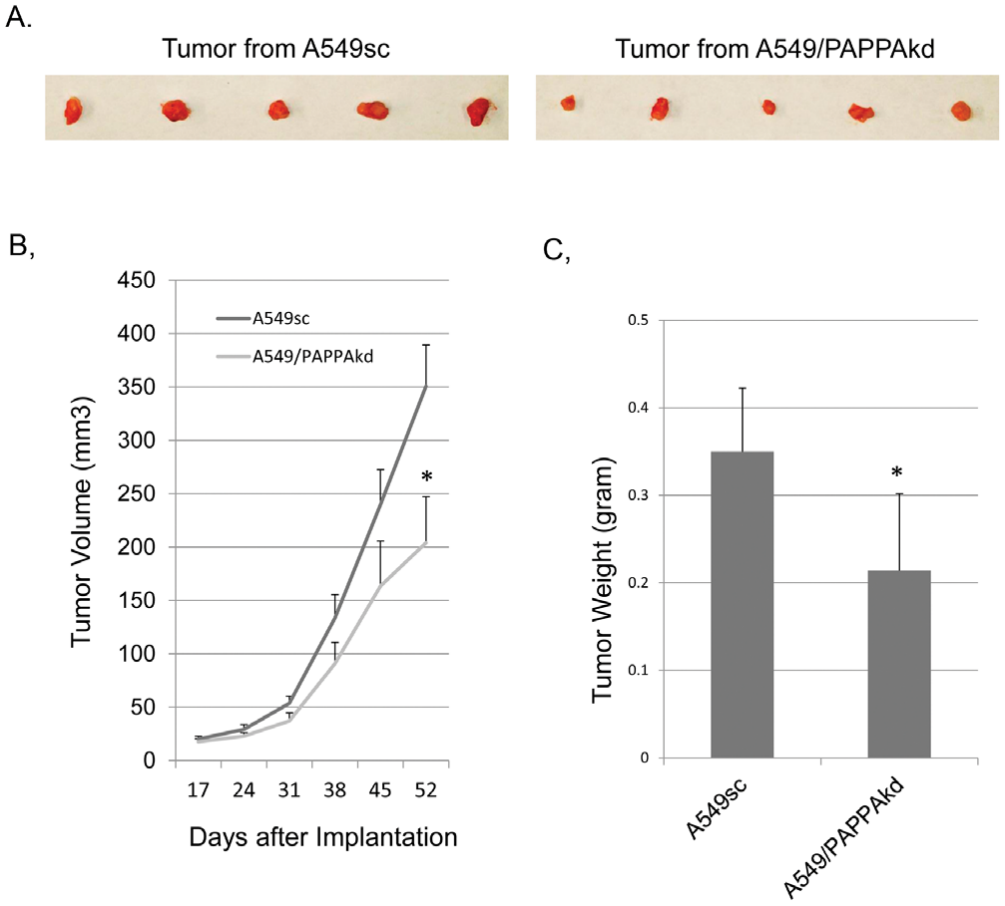
Effect of PAPPA down regulation on xenograft tumor growth. (A) Image of tumor size at the time of dissection. (B) Growth curve of xenograft tumor. PAPPA knockdown A549 cells line A549/PAPPAkd and non-silencing control A549sc were injected subcutaneously into 4 weeks old *nu/nu* athymic mice as described in the Materials and Methods. Results were expressed as mean tumor volume (mm^3^) ± SD (n = 5). (C) Wet tumor weight at the time of dissection. *: *p*<0.05 (student *t* test).

**Figure 4:**
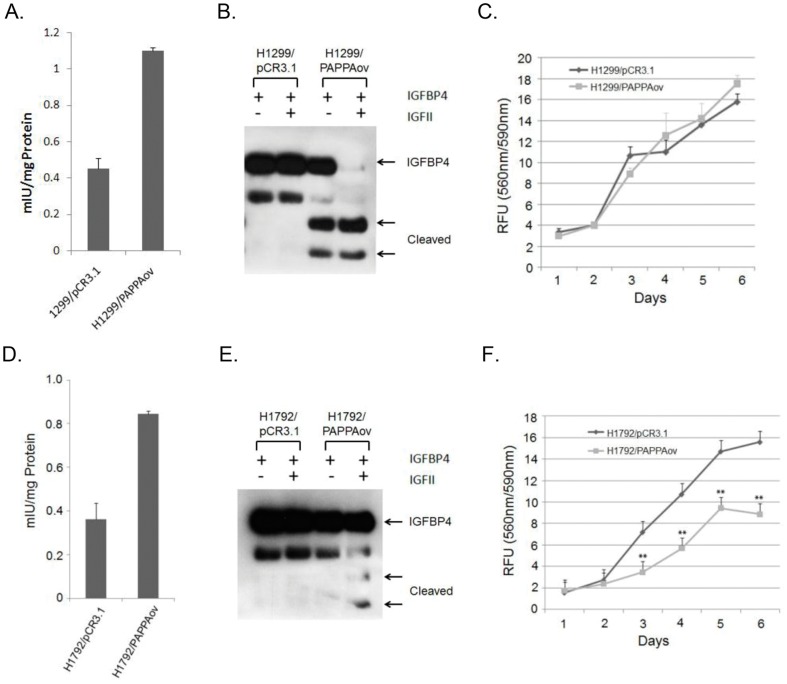
Role of PAPPA on growth of lung cancer cells *in vitro*. (A) Protein levels of PAPPA in H1299 cells over-expressing PAPPA. PAPPA levels in protein extracts from H1299 cell lines stably transfected with PAPPA expression vector (H1299/PAPPAov) and empty control vector (H1299/pCR3.1) were determined by ELISA kit as described in Materials and Methods. Results shown are Mean ± SE of triplicate determinations. (B) IGF dependent protease activity in conditioned medium from H1299/pCR3.1 and H1299/PAPPAov. (C) Growth curve of H1299/pCR3.1 and H1299/PAPPAov cell lines. Viable cells at different time points were measured by CellTiter-Blue as described in Materials and Methods. Results were expressed as Mean ± SE of triplicate determinations of Relative Fluorescence Unit (RFU) of three independent experiments. (D) Protein levels of PAPPA in H1792 cells over-expressing PAPPA. PAPPA levels in protein extracts from H1792 cell lines stably transfected with PAPPA expression vector (H1792/PAPPAov) and empty control vector (H1792/pCR3.1) were determined by ELISA kit as described in Materials and Methods. Results shown are Mean ± SE of triplicate determinations. (E) IGF dependent protease activity in conditioned medium from H1792/pCR3.1 and H1792/PAPPAov. (F) Growth curve of H1792/pCR3.1 and H1792/PAPPAov cell lines.

To assess that the secreted form is important for PAPPA to promote cell growth we generated the stable cell line of A549 transduced with lentiviral particles containing shRNAmir to PAPPA (A549/PAPPAkd) in aim to knockdown the expression and secretion of PAPPA. Over 50% down regulation of PAPPA mRNA has been achieved in clone #82 shRNAmir to PAPPA as confirmed by real-time PCR quantification (Fig. 2A). Importantly, almost all secretion of PAPPA from A549/PAPPAkd cells was abolished as evident by the lack of protease activity in conditioned medium (Fig. 2B). PAPPA is known to degrade IGFBPs and increase the bioavailability of IGFs *in vivo*. It is of great interest to know whether reduction of PAPPA secretion would affect cell growth *in vitro* in cell culture. The effect of PAPPA knockdown on A549 cell growth was evaluated first *in vitro* in comparison with no silencing shRNAmir control cells. No notable change on cell proliferation and growth of A549/PAPPAkd was observed *in vitro* in cell culture condition (Fig. 2C). Failure to detect the effect of PAPPA knockdown on cell growth suggests that cell culture *in vitro* might not be the right model system because it does not have a proper environment for PAPPA to act as protease to increase the bioavailability of IGFs and cells are constantly exposed to many free growth factors including IGFs in culture medium. We decided to examine the role of PAPPA in tumor growth *in vivo* in xenograft models. As expected, tumors generated from A549 cell line with PAPPA knockdown (A549/PAPPAkd) exhibited significant reduction in tumor growth when compared to A549 control cell line (Fig. 3A and B). The average tumor wet weight of A549/PAPPAkd at the end of experiment was 214 mg, which is significantly lower than 360 mg for the control group (Fig. 4C). Taken together, these results indicate PAPPA secreted from tumor cells plays an essential role in promotion of lung cancer cell proliferation and progression *in vivo*.

### Secretion of PAPPA over-expressed in cancer cells promotes tumor growth in xenograft model, but not in vitro in cell proliferation

To further evaluate the role of PAPPA expression in lung cancer development, H1299 and H1792 lung cancer cell lines that showed a low level of endogenous PAPPA expression, were selected for over-expression of PAPPA. Both H1299 and H1792 cells with stably transfected PAPPA expression vector (H1299/PAPPAov and H1792/PAPPAov) achieved about two fold increase in PAPPA protein level when compared to their parental vector control cells (H1299/pCR3.1 and H1792/pCR3.1) (Fig. 4A and D). Importantly, conditioned medium from H1299/PAPPAov cells was able to cleave IGFBP4 substrate in an IGF2 dependent manner while no protease activity was detected in parental vector control cells, suggesting that PAPPA was able to be secreted from H1299/PAPPAov cells and was functionally active (Fig. 4B). Under the same assay condition, conditioned medium from H1792/PAPPAov cells caused little degradation of IGFBP4 substrate, suggesting lack of PAPPA secretion from H1792/PAPPAov cells (Fig. 4E).

The effect of PAPPA over-expression on cell growth was then evaluated in both H1299/PAPPAov and H1792/PAPPAov cells. No notable change of cell growth was observed in H1299/PAPPAov cells when compared to their parental vector control cell, again probably due to the lack of proper microenvironment in cell culture condition as described above (Fig. 4C). Interestingly, H1792/PAPPAov cells showed a significant reduction of cell growth when compared with its vector control cells (Fig. 4 F), suggesting that increases of non-secretive, intracellular PAPPA may exert distinct roles in cancer cell growth.

**Figure 5:**
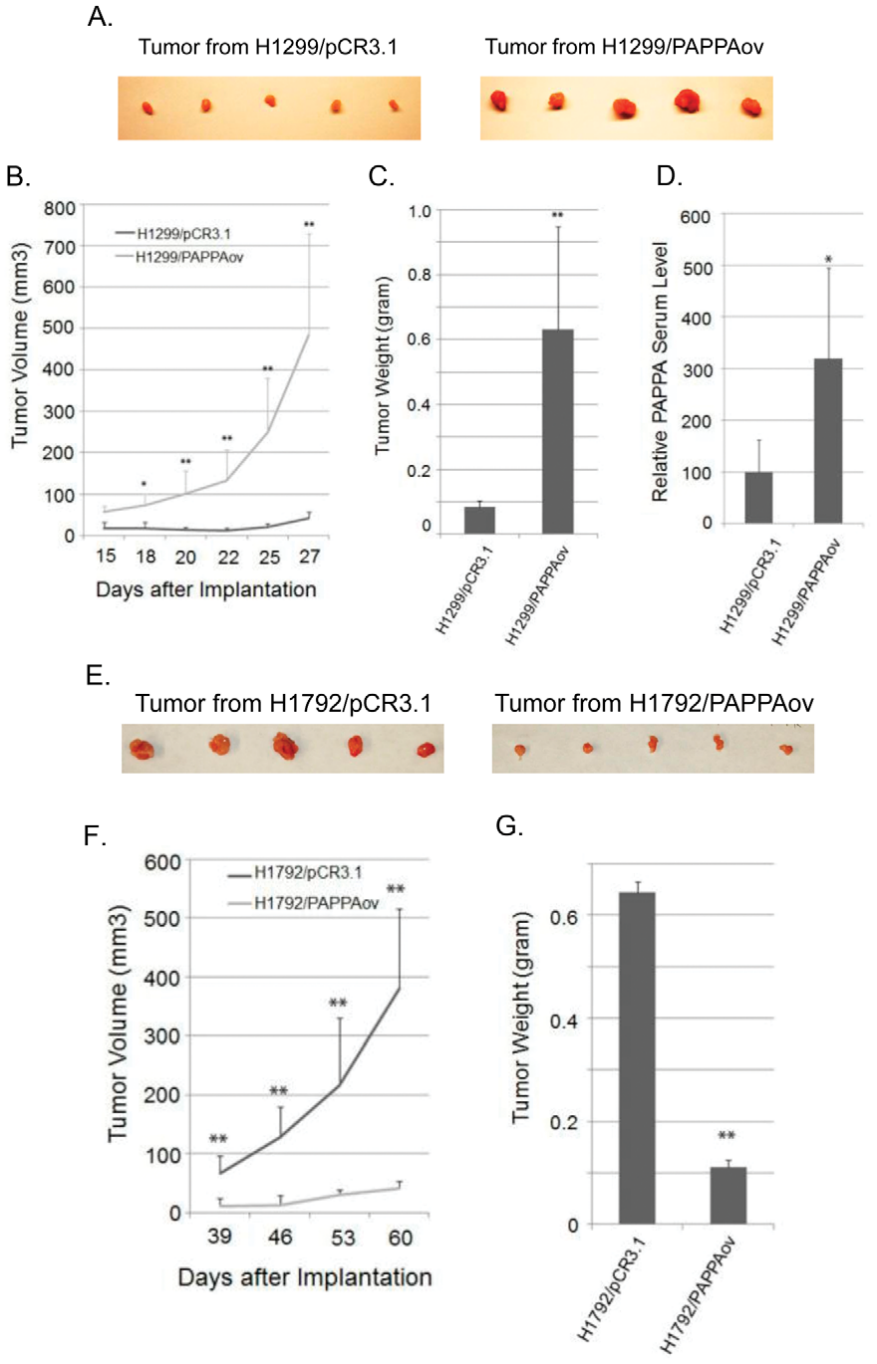
Effect of PAPPA expression on xenograft tumor growth. (A) Image of tumor size from H1299 cells at the time of dissection. (B) Growth curve of xenograft tumor. H1299/pCR3.1 and H1299/PAPPAov cells were injected subcutaneously into 4 weeks old NOD/SCID females. (C) Wet tumor weight at the time of dissection. (D) PAPPA serum level of tumor bearing mice determined by ELISA at the end of experiments. (E) Image of tumor size from H1792 cells at the time of dissection. (F) Growth curve of xenograft tumor. H1792/pCR3.1 and H1792/PAPPAov cells were injected subcutaneously into 4 weeks old *nu/nu* athymic mice. (G) Wet tumor weight at the time of dissection. Tumor sizes were measured and calculated as described in the Materials and Methods. Results were expressed as mean tumor volume (mm^3^) ± SD (n = 5). *: *p*<0.05; **: *p*<0.01 (student *t* test).

Similar to PAPPA knockdown in A549 cells, the effect of PAPPA over-expression was evaluated in a xenograft model. H1299/PAPPAov cells that over-express and secret PAPPA, and H1792/PAPPAov cells that over-express PAPPA without PAPPA secretion were injected subcutaneously into immune-deficient NOD/SCID or *nu/nu* athymic female mice, respectively. As shown in Figure 5, tumors generated from PAPPA over-expression cell line H1299/PAPPAov grew much faster than its vector control line (Fig. 5A and B). Twenty-seven days after inoculation, the average wet weight of H1299/PAPPAov tumors was 630 mg while that of H1299 control was 84 mg (Fig. 5C). The serum level of PAPPA from tumor bearing mice at the end of experiment was measured in both the H1299/PAPPAov group and the respective control group. PAPPA serum level was significantly elevated in H1299/PAPPAov group (Fig. 5D). In contrast to H1299/PAPPAov cells, xenograft tumors from H1792/PAPPAov cells grew much slower than the vector control line (Fig. 5E and F). Sixty days after inoculation, the average wet weight of H1792/PAPPAov tumor was 112 mg, significantly less than the 644 mg of tumor weight derived from H1792/pCR3.1 control cells (Fig. 5G). The results of PAPPA over-expression and down regulation in the lung cancer cell lines were summarized in Table 1. Taken together, these results suggest that secreted form of PAPPA is critical for its tumor promotion activity *in vivo*.

**Table 1:** Summary of PAPPA over-expression and down-regulation experiments.

Cell line	PAPPA Protein level	PAPPA secretion	Tumor growth *in vivo*
A549/PAPPAkd	Decreased	No	Decreased
H1299/PAPPAov	Increased	Yes	Increased
H1792/PAPPAov	Increased	No	Decreased

### Enhancement of AKT signal transduction in increased tumor growth by PAPPA

**Figure 6:**
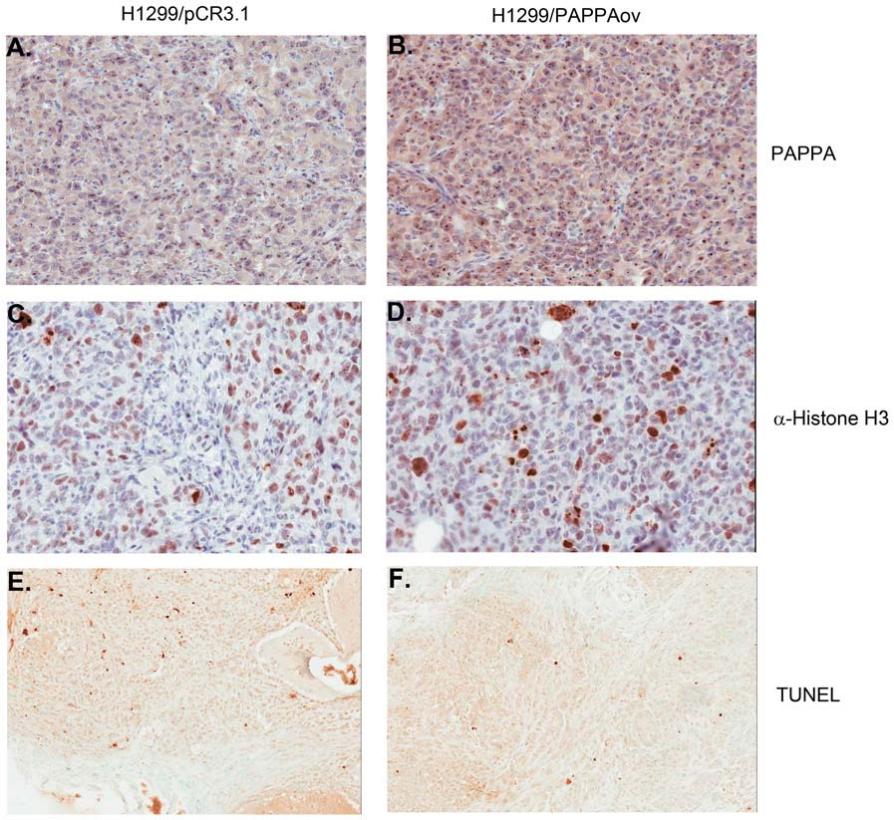
Immunostaining of xenograft tumors from H1299 cells for PAPPA expression (A and B), cell proliferation (C and D with histone H3 antibody) and apoptosis (E and F with TUNEL). A, C and E: xenograft tumors from H1299/pCR3.1 cells; B, D and F: xenograft tumors from H1299/PAPPAov.

**Figure 7:**
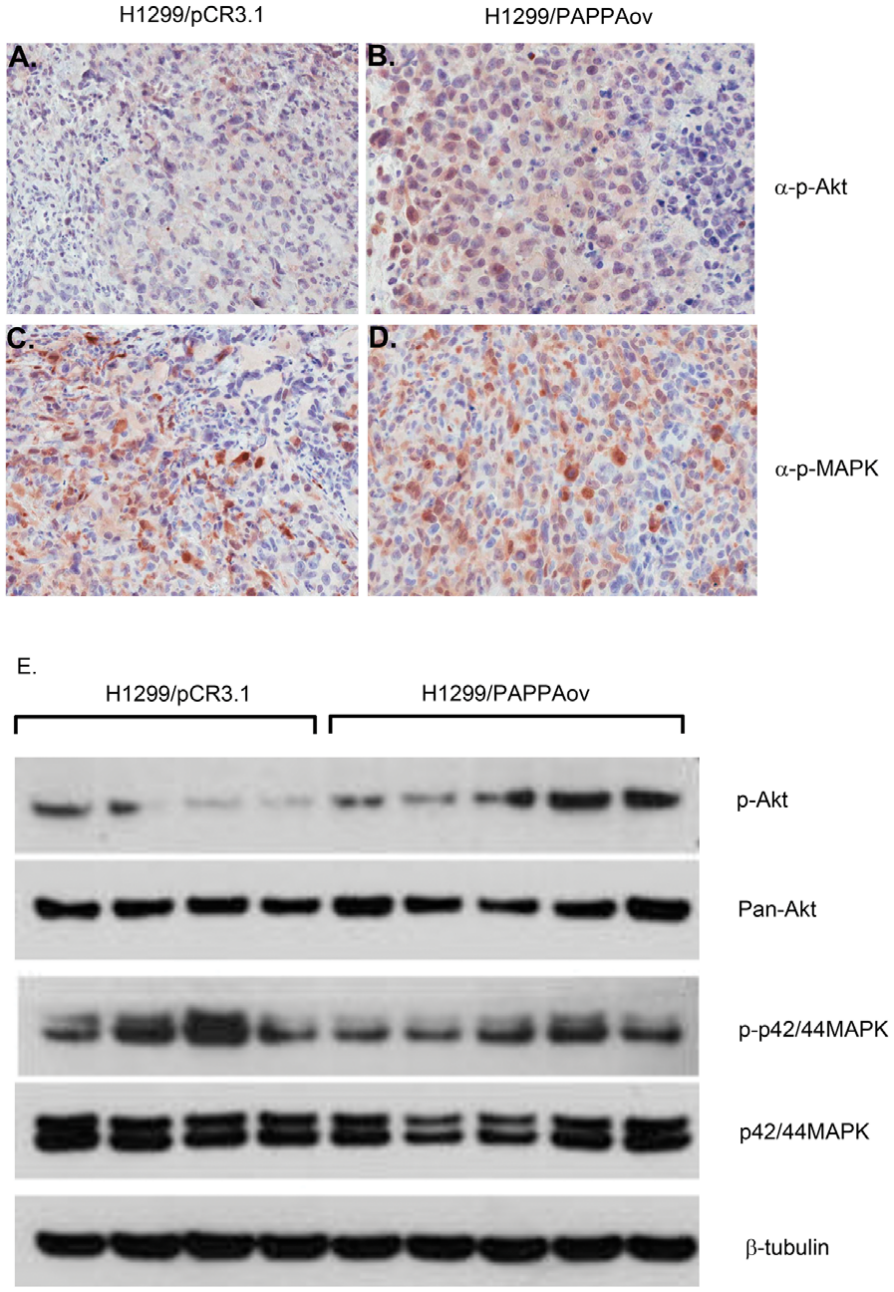
Evaluation of IGF signal transduction in xenograft tumor over-expressing PAPPA. Left panel: Immunostaining for phosphor-Akt (A and B) and phosphor-p42/44 MAPK (C and D) on tumor tissue from H1299/pCR3.1 cells (A and C) and from H1299/PAPPAov cells (B and D). Right panel: western blot quantification of proteins related to IGF signal transduction pathway: P-Akt and P-p42/44 MAPK on protein extracts from tumor tissues. P-: phosphor-protein.

Increased tumor growth in PAPPA secreting cell lines could result from the enhanced IGF signaling caused by degradation of IGFBP4 and thus increases in the bioavailability of IGFs. To test this hypothesis, tumor proliferation and apoptosis were examined using histone H3 as a mitotic marker and TUNEL assay for apoptosis. Significantly more mitotic cells were observed in tumor tissues from H1299/PAPPAov cells than those from H1299/pCR3.1 controls. As for apoptosis, fewer TUNEL positive cells were observed in tumors from H1299/PAPPAov cells than those from H1299/pCR3.1 controls (Fig. 6). To determine possible signal transduction pathways predominantly responsible for tumor growth promotion, the status of phosphor-Akt and phosphor-p43/44 MAPK kinase were examined. As shown in Figure 7, significant greater amount of phospho-Akt positive cells were observed in tumor sections from H1299/PAPPAov cells than tumors from H1299/pCR3.1 control cells. This result was further confirmed by western blot analysis using protein directly extracted from tumor tissues of these two groups. The amount of phosphor-Akt was significantly increased in protein extracts from H1299/PAPPAov tumor samples than those from H1299/pCR3.1 control cells. No notable difference was observed of Phospho-p42/44 MAPK status between H1299/PAPPA and their counterparts.

## Discussion

Our results indicate that expression of PAPPA in lung cancer cell lines varies greatly. The intracellular level of PAPPA protein appears higher in immortalized normal human bronchial epithelial in general than most of the lung cancer cells examined, which appears consistent with the report that the PAPPA chromosomal locus is associated with high frequency of loss of heterozygosity in ovarian tumors [25]. Over-expression of PAPPA in two different lung cancer cells, H1299 and H1792 at similar protein level, however, resulted in different outcomes. Over-expression of PAPPA in H1299 has no notable effect on cell growth *in vitro* but significantly increases in the tumor growth *in vivo* as a xenograft model. In contrast, over-expression of PAPPA in H1792 caused notable reduction of cell growth both *in vitro* and *in vivo* as a xenograft model. By checking both over-expression cell lines we found that H1299 secrets functional PAPPA in conditioned medium while H1792 did not. Even though H1299 and H1792 might have different genetic makeup that may render them reliant on different signaling transduction pathways for growth, it appears that the activity of PAPPA in the promotion of tumor growth is tied to the capability of cells to secret functional PAPPA. Consistent with the role of PAPPA secretion in tumor growth, PAPPA knockdown in A549 resulted in a significant reduction in tumor growth when the cells were implanted in immune-deficient mice. The molecular mechanism(s) underlying the inhibitory effect of intracellular PAPPA in growth of H1792 cells remains to be defined.

PAPPA functions as an IGFBP protease to control the local concentration of free active IGFs available to their receptors by IGFBP degradation and to indirectly enhance the IGF signaling pathway. Our *in vitro* and *in vivo* results appear in consistent with this model. PAPPA secretion changes in both H1299 and A549 has no notable effect on cancer cell growth *in vitro* in culture, due to the fact that cell culture lacks of a proper microenvironment where IGFs in culture medium are in free access to their receptors. Xenograft models proved to be more appropriate to evaluate the effect of PAPPA secretion on tumor growth. We found that the increased PAPPA secretion in tumor tissues is accompanied predominantly by activation of IGF1R-Akt signaling pathway. In addition, increased cell proliferation and reduced apoptosis were observed in tumor tissues over-expressing PAPPA. Our results indicate that deregulation of PAPPA expression and secretion is one of the driving forces for tumor progression.

A variety of reagents have been developed to modulate IGF signaling activities including monoclonal antibodies against IGFs and receptor IGF1R, as well as associated RTK inhibitors in aim for cancer treatment [31]. IGF-1R interacts with insulin receptors (IRs) to form heterotetramers that mediate insulin activities. The high homology between IGFIR and IR has made it difficult to develop RTK inhibitors that specifically block IGF signaling [32], [33]. Reagents that interfere with insulin activities inevitably cause the adverse effect of hyperglycemia. In fact, many reagents targeting this pathway showed hyperglycemia in clinical trials [31]. The role of PAPPA in tumor progression defined in this report revealed a new potential target related to targeted therapy of the IGF signaling pathway. During the course of our study, PAPPA was over expressed in the SKOV3 ovarian cancer cell line by Boldt HB and Conover CA [34] and they reported a tumor promotion effect of PAPPA. Our study confirmed the role of PAPPA in lung tumor growth and further defined that it is the secreted PAPPA from tumor cells that is critical for its tumor promotion activities. Higher serum level of PAPPA was observed in mice implanted with cancer cells over-expressing PAPPA when compared with control cells. It is reasonable to hypothesize that increased serum level in cancer patients may correlated with enhanced IGF signaling in tumor cells. Further study to test this hypothesis could lead to the establishment of PAPPA as a biomarker for IGF targeted therapy. In addition, it has been reported that high serum level of PAPPA are adversely related to atherosclerotic plaques in human coronary arteries. It is conceivable that PAPPA neutralizing antibody or its specific protease inhibitor would have beneficial effect on cancer therapy with additive effect of a healthier heart.
